# The effects of Leptin and Adiponectin on Pdx1, Foxm1, and PPARγ Transcription in Rat Islets of Langerhans

**DOI:** 10.5812/hepatmon.9055

**Published:** 2013-06-22

**Authors:** Mandana Mahmoodzadeh Sagheb, Negar Azarpira, Mokhtar Mokhtary, Sayyed Ebrahim Hosseini, Ramin Yaghobi

**Affiliations:** 1 Department of Biology, Science and Research Branch, Islamic Azad University, Shiraz, IR Iran; 2 Transplant Research Center, Shiraz University of Medical Sciences, Shiraz, IR Iran; 3 Department of Biology, Kazeroon Branch, Islamic Azad University, Kazeroon, IR Iran

**Keywords:** Leptin, Adiponectin, PPAR gamma, Islets of Langerhans, Insulin

## Abstract

**Background:**

Leptin and adiponectin are two hormones, which are released from adipocytes in order to control energy expenditure. Both hormones are also involved in glucose homeostasis through control of insulin secretion from pancreatic islets. Since Pdx1, PPARγ, and foxm1 play important roles in islets function, it is essential to understand how these genes are regulated in the islets of Langerhans.

**Objectives:**

We have designed an experiment to identify the effect of leptin and adiponectin treatment on Pdx1, PPARγ, and foxm1 transcription.

**Materials and Methods:**

Islets were isolated from adult male rats by collagenase and incubated with different concentrations of leptin and adiponectin for 24 hours. Next, by means of real time PCR, we evaluated the gene transcription related to a housekeeping gene. The effect of leptin and adiponectin on insulin secretion was evaluated by ELISA.

**Results:**

Leptin decreased PPARγ transcription and insulin secretion, while adiponectin significantly increased Pdx1 and PPARγ transcription and insulin secretion in rat islets. The transcription of foxm1 did not change in the islet cells treated with leptin or adiponectin.

**Conclusions:**

These findings indicate the possibility that Pdx1 and PPARγ transcription is a mediator of leptin and adiponectin function in control of insulin secretion and glucose homeostasis in pancreatic islets.

## 1. Background

Control of pancreatic islets function is required for regulation of plasma glucose homeostasis. Insulin secretion by beta cells is adapted to the body’s need and insulin demands and depends on nutritional status and hormonal as well as neural signals (1). Leptin and adiponectin are adipokines which regulate insulin sensitivity and energy homeostasis (2). Leptin decreases insulin sensitivity, while adiponectin enhances it (3). The plasma level of leptin is proportional to body fat content and Body Mass Index (BMI) (4, 5). However, unlike leptin, adiponectin systemic concentration is negatively related to the adiposity (6). Leptin deficiency in *ob*/*ob* mice and leptin receptor deficiency in *db*/*db* mice led to hyperinsulinemia even before the progression of obesity and diabetes and this hyperinsulinemia was ameliorated by administration of recombinant leptin to the *ob*/*ob* mice. These results suggest the direct inhibitory action of leptin on insulin secretion from pancreatic β-cells (7-9). Additionally, leptin receptor mRNA, extracted from rat islets was even greater than that found in the brain, and was also detected in the pancreatic β-cell line (7, 10). Furthermore, it has been reported that adiponectin influenced glucose induced insulin secretion because its receptors were identified in the pancreatic islet cells (11). The pancreatic-duodenal homeobox factor1 (Pdx1) is a key transcription factor which regulates early pancreas formation. Moreover, it controls several aspects of mature β cell function, including glucose-mediated insulin secretion in adult islet beta cells (12). Reduced Pdx1 expression in the β cell occurs in glucose toxicity and accompanies beta cell failure (13). However, the signaling pathway responsible for glucose induced insulin gene transcription is not fully understood. We hypothesized that the inhibitory action of leptin or the stimulatory effect of adiponectin on insulin secretion might occur through alteration in Pdx1 transcription. The nuclear hormone receptor, peroxisome proliferator-activated receptor γ (PPARγ), is involved in the regulation of insulin sensitivity and glucose homeostasis (14). Two thiazolidinediones (TZDs), rosiglitazone and pioglitazone, and agonists of PPARγ, in addition to their influence on peripheral insulin-sensitive tissues, may also be effective in endocrine pancreas. PPARγ transcription has been detected in islet cells as well as clonal beta-cell lines (15). Recently, impaired insulin secretion has been reported in heterozygous PPARγ-deficient mice, which is related to increased islet triacylglycerol content (16). However, whether PPARγ agonists can directly influence the β-cell function remains unclear. It has been reported that the TZD, troglitazone, increase the amount of insulin secretion from isolated islets and HIT-T15 cells (17). Thus, we aim to determine whether leptin and adiponectin as two potent regulators of insulin secretion in islets can influence the transcription of PPARγ as their target. FoxM1 is another transcription factor which regulates the expression of several cell cycle genes and is required for the maintenance of adult beta-cell mass, beta-cell proliferation, and glucose homeostasis (18). Foxm1 knock out in mice did not cause any abnormality in their pancreas at birth; however, their β-cell mass gradually declined with age (18). Diabetes also resulted in impaired islet function and defect in postnatal β-cell mass expansion (18). β-cell proliferation occurs in adult obese humans in order to increase β-cell mass to compensate for insulin resistance; FoxM1 expression is critical in this process (17). The possibility that leptin has a regulatory effect on Foxm1 expression is further supported by non diabetic C57BL/6 leptin (ob/ob) mouse model since these animals developed up regulation of islets Foxm1 (17).

## 2. Objectives

Here, we have designed an *in vitro* experiment to investigate the role of leptin and adiponectin on FoxM1 transcription in the islets of Langerhans.

## 3. Materials and Methods

### 3.1. Islets Isolation

Adult Wistar male rats weighing 300-350 g were maintained under 12 h light, 12 h dark condition at 22°C. Animals had free access to pelleted food and tap water. All animal experiments were conducted in accordance with the ethical standards of the Animal Care and Use Committee of Shiraz University of Medical Sciences, Shiraz, IR Iran. Anesthesia was provided by Ketamin (100 mg/kg ip). Rats islets of Langerhans were isolated by collagenase P (Roche Applied Science, Germany). The pancreatic duct was cannulated with PE50 tube (Becton Dickinson Company, USA) and collagenase P (15 mg in 15cc HBSS (Hank’s Balance Salt Solution)) was injected in to the duct. Distended pancreas was kept at 37⁰C for 25 min. The islets were then washed by medium A [HBSS, 1% HEPES (Sigma-Aldrich, USA), 2% Fetal Bovine Serum (FBS) (Sigma-Aldrich, USA)] for 5 times, isolated, and purified from exocrine cells by means of lymphoprep (Axis-Shield, Norway). Islets size and purity were determined by microscopic sizing on a grid after staining with 1, 5-diphenylthiocarbazone (DTZ) (Merck Millipore, Germany). Next, the islets were incubated in RPMI (Sigma-Aldrich, USA) at 37⁰C supplemented with 10% fetal bovine serum and 1% penicillin-streptomycin for 24 h before use.

### 3.2. Cell Culture

To investigate whether the transcription of Pdx1, FoxM1, and PPARγ genes could be directly regulated by leptin and adiponectin, the islets were seeded into a 48-well plate at 100 islets per well and incubated at 37⁰C in an atmosphere of 5% CO_2_ in RPMI supplemented with 10% fetal bovine serum and 1% penicillin-streptomycin (Roche Applied Science, Germany) for 24 h before use.

### 3.3. Islets Treatment With Leptin and Adiponectin 

After 24 h of incubation, the medium from each well was subsequently replaced by medium alone or medium containing 3.125, 6.25, 12.5, 25, and 50 nmol/l leptin (Sigma-Aldrich, USA) or adiponectin (Sigma-Aldrich, USA) at doses of 2.5, 5, and 10 µg/ml and the plate was incubated for 24 h.

### 3.4. RNA Isolation and CDNA Synthesis

Total RNA was extracted from the islets and the cell line by RNA kit II (Invitek, Germany) according to the manufacturer’s instructions. The extracted RNA was quantitated by OD_260/280_ measurement. Next, the total RNA (10 μg) of cell culture extracts was reverse transcribed in a 20-μl volume using random hexamer primers with enzyme and buffers supplied by the cDNA First Strand Synthesis kit (Fermentas, Life Science, EU (Germany)).

### 3.5. Quantitative Real Time PCR

First, 5 µg cDNA was added to taq man master mix (TaKaRa, Takara Shuzo, Otsu, Japan). The final volume of the PCR was 20 μl: 10 μl Master Mix, 0.6 μl of each primer, 0.6 μl prob, 0.4 μl reference dye, and 2.8 μl dH2O. Amplification of DNA was performed under the following conditions: 10 min at 95⁰C, 10s at 95⁰C, and 30s at 60⁰C for 40 cycles. Primers and probe used for real-time PCR were selected using rat genomic sequences as templates and NCBI (http://www.ncbi.nlm.nih.gov/pubmed) and Allele ID programs. GAPDH was selected as endogenous control and the transcription of PPAR-γ, PdX-1, and foxm1 was checked in relation to GAPDH (Table 1).

**Table 1.** The Sequence of Primers and Probes

	Forward Primer	Reverse Primer	Probe
**GAPDH**	GGCTCTCTGCTCCTCCCTGTTC	CGGCCAAATCCGTTCACACCGA	GCCGCATCTTCTTGTGCAGTGCCAGCC
**PPARγ**	CAGAGGGACAAGGATTCATGACC	TTCACAGCAAACTCAAACTTAGGC	AGTCACCAAAGGGCTTCCGCAGGC
**Pdx-1**	CCGAGCTTCTGAAAACTTTGAGG	TGGGAGCCTGATTCTCTAAATTGG	TGCCTCTCGTGCCATGTGAACCGC
**FoxM1**	CAAGGTAAAAGCCACGTCTAAGC	GGAGCAGCAGGTGACTAATGG	TGGGCATTTCCTGGTCCTCACGGC

### 3.6. Insulin Secretion in Vitro

The effect of leptin on insulin secretion was assessed by static incubation of the islets. Groups of 20 islets (hand-picked) were placed in 6 well plates and incubated in Krebs-Ringer-HEPES (KRH) buffer (125 mM NaCl, 4.74 mM KCl, 1 mM CaCl_2_, 1.2 mM KH_2_PO_4_, 1.2 mM MgSO_4_, 5 mM NaHCO_3_, 25 mM HEPES, 0.1% BSA, pH 7.4) containing 2.8 mM glucose (Sigma-Aldrich, USA). After 1 h of pre-incubation, supernatant was collected and the cells were stimulated with KRH buffer containing 8.3 mM glucose for 1 h. Again, the supernatant was collected. This procedure was repeated with KRH buffer containing 16.7 mM glucose. To test the effects of leptin and adiponectin, these peptides were applied during the 1 h incubation period at the given glucose concentration. We used 0.2 and 2 nmol/l of leptin because in previous studies, 2 nmol/l of leptin was the most effective concentration of this hormone in rat islets and higher concentrations were almost ineffective (7). Doses of 2.5 and 5 µg/ml of adiponectin were selected for treating the islets (11). Insulin in the supernatant was determined by rat insulin ELISA kit (Mercodia, Sweden).

### 3.7. Cell Apoptosis 

Because we incubated the islets with both physiological and pharmacological doses of leptin and adiponectin, in order to examine whether the cells were still alive, we checked them for apoptosis. Groups of 20 islets were treated with leptin (3.125 and 50 nmol/l) and adiponectin (5 and 10 µg/ml) for 24 hours. After incubation at 37C°, apoptosis of the samples was determined by an *in situ* cell death detection kit (POD) (Roche Applied Science, Germany). Cleavage of DNA during apoptosis may yield double-strands as well as single-strand breaks. The principle of this technique is labeling of DNA strand breaks by deoxynucleotidyl transferase (TdT) which catalyzes the polymerization of labeled nucleotides to free 3’ OH DNA ends in a template-independent manner termed the TUNEL-reaction. After substrate reaction, stained cells can be analyzed under the light microscope. The percentage of apoptotic cells was determined by counting the apoptotic (brown) cells relative to the live (blue) ones. The experiment was done in triplicate.

### 3.8. Statistical Analysis

The data are shown as mean ± SD. For evaluation of each gene transcription, 3-5 test groups were compared with the controls and each experiment was repeated 3 times. Different groups were compared through one-way ANOVA followed by Tukey’s test for pair wise comparison. For evaluation of insulin secretion, the study groups were compared by two-way ANOVA. P < 0.05 was considered as statistically significant. The statistical analyses and design of the graphs were performed using the Graph Pad Prism 5 software.

## 4. Results

**Figure 1.**
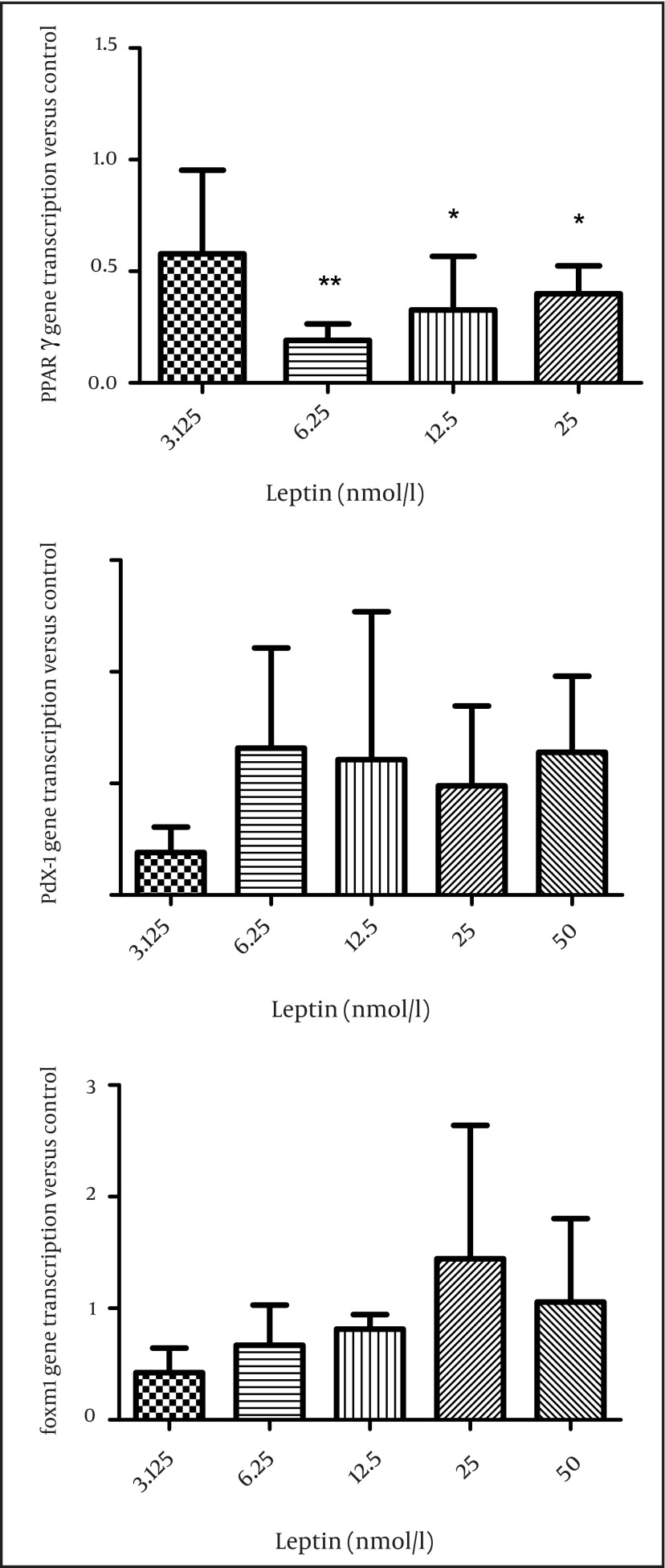
The Effect of Different Concentrations of Leptin on Gene Transcription (Pparγ, Pdx1, and Foxm1) in Rat Islets of Langerhans The effect of different concentrations of leptin incubation (3.125, 6.25, 12.5, 25, and 50 nmol/l) for 24 hours on pparγ transcription (A) on pdx1 transcription (B) on foxm1 transcription (C) in rat islets (n = 6). All Data are shown relative to the controls (leptin 0). The data are shown as mean ± SD with triplicates for rat islets.*, P < 0.05 and **, P < 0.01 for each value versus its control without leptin; Analysis (A-C) by one-way ANOVA followed by Tukey’s test.

In order to evaluate the mechanism of leptin action on insulin secretion, first we studied the effect of leptin on the transcription of PPARγ, pdx-1, and foxm1 in the isolated islets of Langerhans. Leptin decreased PPARγ transcription and this was significant at 6.25 (P < 0.01), 12.5, and 50 nmol/l compared to the control group (P < 0.05) (Figure 1A). The transcription of Pdx-1 and foxm1 did not change significantly after incubation of the islets with leptin (Figure 1B, C).

### 4.1. The Effect of Leptin on Insulin Secretion in the Islets of Langerhans

**Figure 2.**
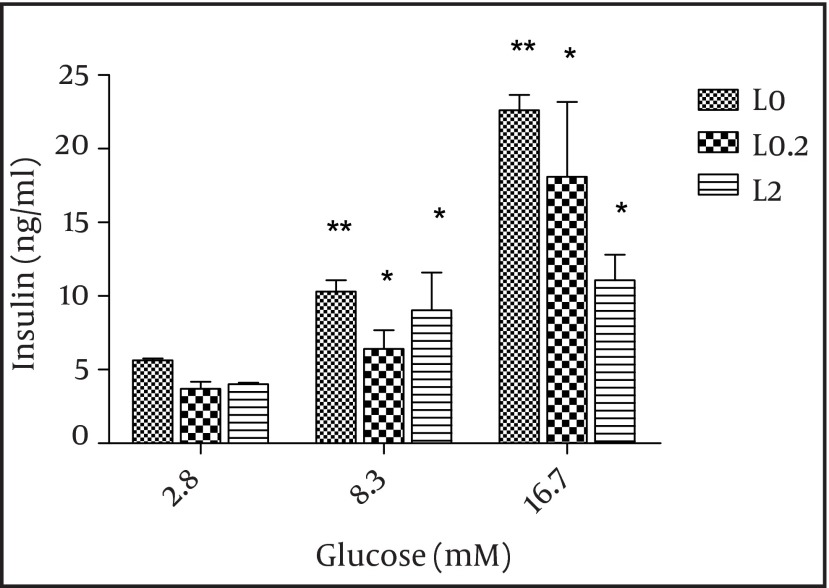
The Effect of Leptin on Insulin Secretio Static 1 hour incubation with leptin significantly decreased insulin release at glucose levels of 8.3 and 16.7 mM in vitro. The results from systems with leptin (0.2 and 2 nmol/l) are shown in grey and black and those from systems without leptin (control) are shown in white. The data shown are mean ± SD with triplicates for rat islets. * P = 0.06 and ** P < 0.01 for each value versus its control (glucose concentration 2.8 mM and without leptin); Analysis by two-way ANOVA (n = 9).

**Figure 3.**
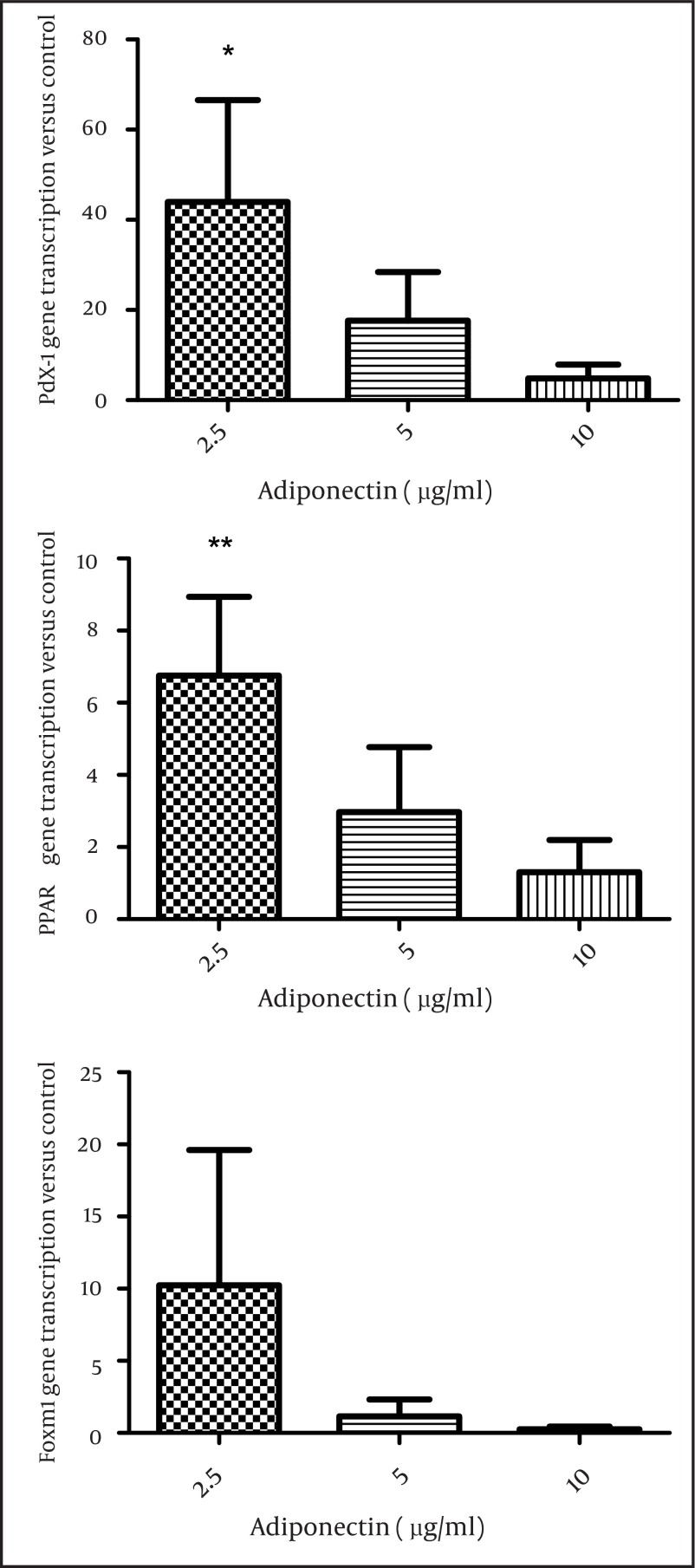
The Effect of Different Concentrations of Adiponectin on Gene Transcription (PPARγ, Pdx1, FoxM1) in Rat Islets of Langerhans The effect of adiponectin incubation (2.5, 5 and 10 µg/ml) for 24 hours on PPARγ transcription (A), on pdx1 transcription (B), on FoxM1 transcription (C) in rat islets (n = 6). All Data are shown relative to the controls (Adiponectin 0). The data are shown as mean ± SD with triplicates for rat islets.*, P < 0.05 and **, P < 0.01 for each value versus its control without adiponectin; Analysis (A-C) by one-way ANOVA followed by Tukey’s test.

Then, we applied the two effective leptin concentrations to the isolated islets culture plate to check the islets for insulin secretion. Stimulation of the islets with 8.3 and 17.6 mM glucose significantly elevated insulin secretion by these cells (P < 0.01) and both 0.2 and 2 nmol/l of leptin decreased insulin secretion in comparison to the control group (P = 0.06) (Figure 2). We also studied the effects of adiponectin treatment on the transcription of PPARγ, pdx-1, and foxm1 in the islet cells. Adiponectin increased the transcription of PPARγ (P < 0.01) (Figure 3E).

Additionally, we observed that the transcription of pdx1 increased (P < 0.05), while the transcription of foxm1 did not change significantly in the islet cells after 24 hours treatment with adiponectin (Figure 3D, F).

### 4.2. The Effect of Adiponectin on Insulin Secretion in the Islet Cells

**Figure 4.**
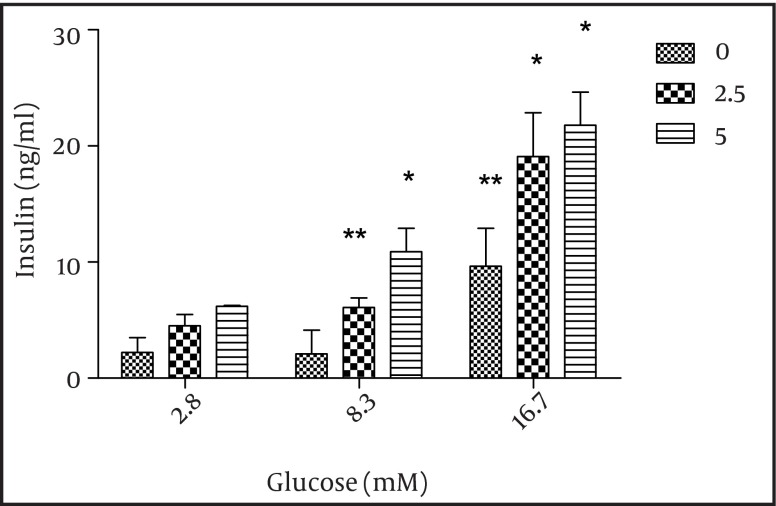
The Effect of Adiponectin on Insulin Secretion Static 1 hour incubation with adiponectin significantly potentiated insulin release at glucose levels of 8.3 and 16.7 mM in vitro. The results from the systems with adiponectin (2.5, 5 µg/ml) are shown in grey and black and those from the systems without adiponectin (control) are shown in white. The data are shown as mean ± SD with triplicates for rat islets. * P = 0.06 and ** P < 0.01 for each value versus its control (glucose concentration 2.8 mM and without adiponectin); analysis by two-way ANOVA (n = 9).

The effect of adiponectin on insulin secretion was assessed by incubation of the islets with 2.5 and 5 µg/ml adiponecin in the presence of 8.3 and 16.7 mM glucose. We found that the two glucose concentrations as well as 2.5 and 5 µg/ml adiponectin significantly increased insulin secretion (P < 0.01 and P < 0.05, respectively) (Figure 4).

### 4.3. The Effect of Leptin and Adiponectin Treatment on Islet Cell Apoptosis

**Figure 5.**
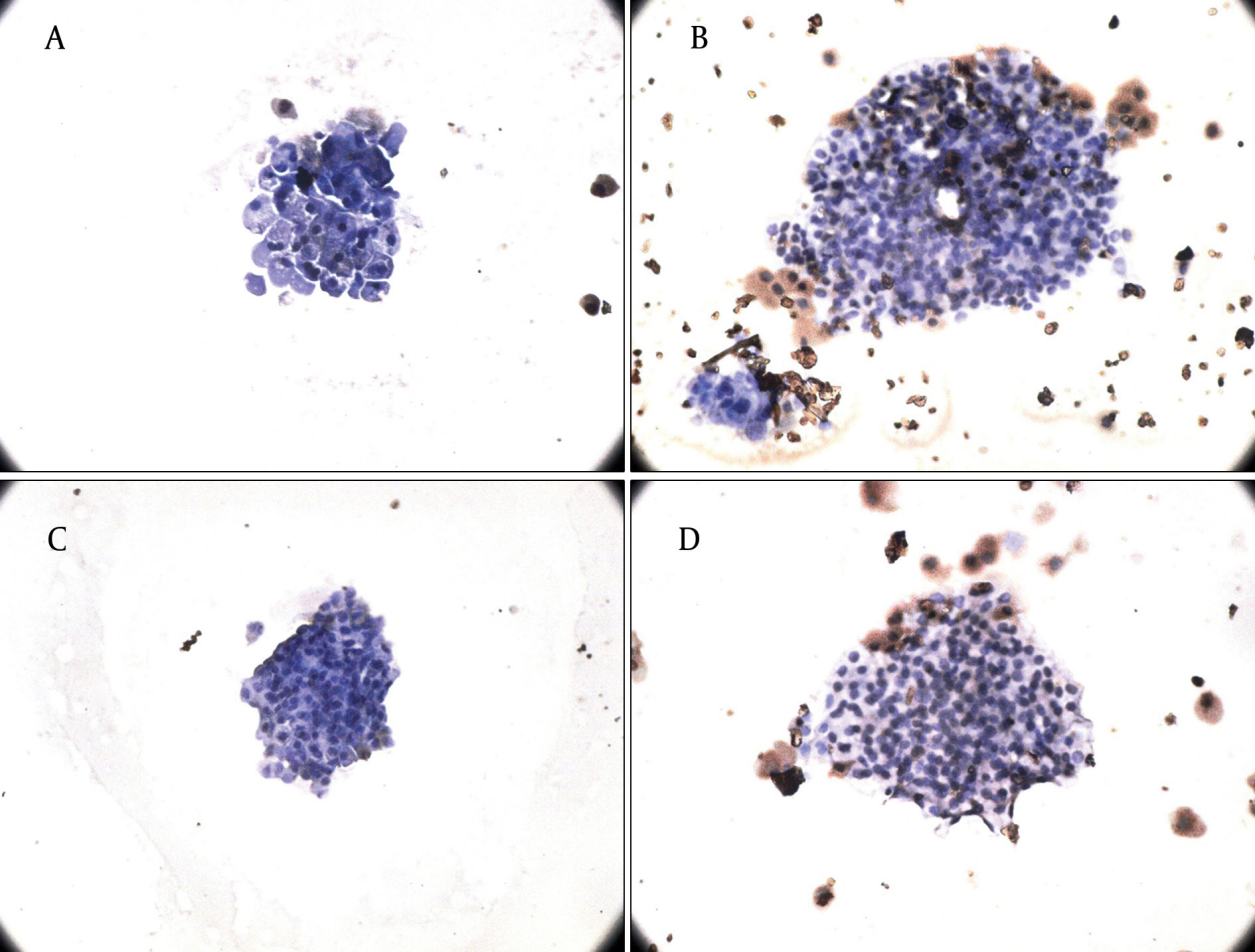
The Effect of Leptin and Adiponectin Incubation for 24 Hours on Apoptosis of Rat Islets of Langerhans A, B) The effect of leptin treatment (3.125 and 50 nmol/l) for 24 hours on apoptosis of rat islets. A) Treatment with leptin 3.125 nmol/l, B) Leptin dose of 50 nmol/l (n = 2). C, D) The effect of adiponectin treatment (5 and 10 µg/ml) for 24 hours on apoptosis of rat islets of Langerhans. C) Adiponectin dose of 2.5 µg/ml, D) Treatment with 10 µg/ml adiponectin (n = 2) (×40).

Finally, we observed that physiological concentrations of leptin and adiponectin were not toxic for the cells and only less than 5% of the islets underwent apoptosis after treatment with pharmacological concentrations of hormones (Figure 5).

## 5. Discussion

Among other functions, leptin contributes to glucose homeostasis both at central (hypothalamus) and peripheral level (pancreatic islets) (19). Adiponectin is also a well-known and important hormone which influences glucose homeostasis (6). Furthermore, rat islets of Langerhans express both leptin (ObRb) (7) and adiponectin receptors (ADR-1,ADP-2) (20) and this is clearly an evidence indicating that beta cells are targets for leptin and adiponectin action. Insulin is regulated in several levels from gene expression to insulin secretion (21). However, the signaling pathways responsible for glucose induced insulin gene transcription are under investigation. Therefore, we have investigated the role of leptin and adiponectin in the transcription of transcription factors related to insulin synthesis as well as insulin secretion. The pancreatic duodenal homeobox domain-1 (Pdx1) is one of these transcription factors (21) which is normally expressed in β-cells and directly regulates the expression of the insulin gene and glucose-stimulated insulin secretion pathway (22). In mature β-cells, Pdx1 stimulates the insulin gene expression and other genes associated with glucose metabolism, such as GLUT2 and glucokinase (16).

In this study, we found that leptin decreased Pdx1 transcription in the islet cells though this decline was not significant. Incubation of the islets with leptin for longer periods of time might result in significant data. The results obtained from Alox5−/− mice which had increased plasma leptin and fasting glucose levels but lower fasting insulin levels suggest a relationship between leptin level and Pdx1 expression. In these animals, insulin secretion and Pdx1 expression in the isolated islets were significantly lower than the wild type ones. Furthermore, deletion of ALOX5 from isolated human islets with siRNA diminished insulin and PDX1 gene expression by 50% and insulin secretion by 3-fold (23). These results propose the inhibitory effect of leptin on Pdx1 transcription. It has been reported that globular adiponectin causes a significant 45% increase in PDX-1 expression (15). We also observed a similar excitatory action for adiponectin on rat islets of Langerhans incubated with 2.5 µg/ml adiponectin. As PPARγ expression has been found in human and rat islets as well as clonal β-cell lines (24), we investigated the consequences of leptin and adiponectin treatment on alterations of PPARγ transcription in rat islet cells. We detected the inhibition of PPARγ transcription by leptin in the islets. Previously, it was demonstrated that PPARγ expression was inhibited by leptin in hepatic stellate cells in the mouse model of liver damage (25) and we detected the same inhibitory action in the islets of Langerhans. The effect of PPARγ agonists on adiponectin expression has been studied in some researches (26); however, here we performed an *in vitro* experiment to evaluate the impact of leptin on PPARγ transcription in rat islet cells and showed that adiponectin increased PPARγ transcription. The expression of foxm1 and its essential role in β-cell proliferation in response to physiological stimuli has been demonstrated in human and mice islets of Langerhans. An *in vivo* experiment showed that obesity-stimulated β-cell proliferation excited foxm1 transcription and lack of proliferation led to diabetes in mice (17). For the first time, we studied the effect of leptin and adiponectin on the transcription of foxm1 in rat islets of Langerhans; however, no significant change was observed in foxm1 mRNA in the islets treated with leptin and adiponectin. These results may represent the lack of a relationship between foxm1 and glucose-stimulated insulin secretion pathway. Of course, further confirmation of these primary results needs the application of a western blotting assay. The role of leptin and adiponectin in insulin secretion was also evaluated in the present study. Thus, we performed an experiment to confirm the role of leptin and adiponectin on insulin secretion. We reported that 0.2 and 2 nmol/l of leptin decreased insulin release from the islets of Langerhans. The leptin levels applied in this study were within the rodent physiological range and were effective concentrations of leptin in suppressing insulin secretion, while higher levels are less efficient (27). This inhibitory action of leptin has been reported in human and rodent isolated islets (8). In this study, we noticed the excitatory role of adiponectin on insulin secretion from rat islets of Langerhans. However, in the literature, there are some controversies about the effect of adiponectin on insulin secretion. In an earlier experiment, for instance, no stimulatory effect of adiponectin on human islets was detected (28). Another research also reported the dual role of adiponectin on insulin secretion in insulin-resistant mice (11). Nevertheless, a recent study showed that globular adiponectin increased insulin secretion from rat islet cells at high glucose concentrations (16.7 mM) (29). In the current study, we detected this promoting role of adiponectin in both 8.3 and 16.7 mM glucose. These findings propose the regulatory role of leptin and adiponectin in insulin secretion and PPARγ and Pdx1 transcription which are associated with glucose induced insulin secretion; however, leptin and adiponectin do not seem to affect foxm1 transcription in rat islets of Langerhans. Therefore, we suggest a western blotting assay to confirm these results.
